# Psychometric Evaluation of Ecological Momentary Assessment Items for Mood in a Non‐Clinical Sample

**DOI:** 10.1002/brb3.71598

**Published:** 2026-07-14

**Authors:** Judith Rohde, Stephanie Homan, Marta A. Marciniak, Steffi Weidt, Erich Seifritz, Stephan T. Egger

**Affiliations:** ^1^ Department of Adult Psychiatry and Psychotherapy University Hospital of Psychiatry Zurich, University of Zurich Zurich Switzerland; ^2^ Department of Psychology University of Zurich Zurich Switzerland; ^3^ Digital Mental Health Management Research Group, Healthy Longevity Center University of Zurich Zurich Switzerland; ^4^ Department of Psychology, Education and Child Studies Erasmus University Rotterdam Rotterdam Netherlands; ^5^ Psychiatric Services St. Gallen University of St. Gallen St. Gallen Switzerland

**Keywords:** digital health, Ecological Momentary Assessment, factor analysis, mood assessment, network analysis, psychometrics

## Abstract

**Background:**

Smartphone‐based Ecological Momentary Assessment (EMA) enables the real‐time measurement of emotional states in daily life, reducing recall bias and capturing clinically meaningful fluctuations. However, evidence regarding the reliability and validity of EMA measures remains limited, and validated instruments are scarce, highlighting the need for EMA‐specific psychometric evaluation.

**Objective:**

To assess the reliability, validity, and structural characteristics of a brief 11‐item smartphone‐based EMA of mood in a non‐clinical sample.

**Methods:**

We used data from a randomized controlled trial evaluating a 1‐week digital self‐efficacy training in a stress‐enriched non‐clinical sample of 93 Swiss university students. Baseline psychometric assessments included the Beck Depression Inventory II (BDI II), the Positive and Negative Affect Schedule (PANAS), the General Self‐Efficacy Scale (GSE), the State and Trait Anxiety Inventory (STAI), and the Perceived Stress Scale (PSS). The EMA assessment included moods such as cheerful, irritated, anxious, happy, insecure, lonely, relaxed, sad, overthinking, focused, and stressed. Analyses included descriptive statistics, internal consistency (Cronbach's alpha), and external validity (correlations between baseline questionnaires and participant‐level aggregated EMA ratings from the first 24 h). Exploratory and confirmatory factor analyses and network analyses assessed the structure.

**Results:**

We considered the data of all 93 participants for the analysis. Participants (78.5% female) were on average 23.27 years of age (*SD* = 3.49). EMA items showed normal distribution, good internal consistency (*α* = 0.88), and low correlations (0.19–0.39) with the BDI II, the PANAS positive affect subscale, and the GSE. Moderate correlations (0.40–0.48) were found with the PANAS negative affect subscale, the STAI, and the PSS. An exploratory factor analysis indicated two or three factors, while network analysis revealed positive and negative affect communities. Confirmatory analysis suggested that the network model showed the most favorable fit indices among the models examined (CFI = 0.99; TLI = 0.99; RMSEA = 0.01).

**Conclusion:**

The smartphone‐based EMA mood item set provided preliminary evidence of reliability and validity in this stress‐enriched non‐clinical student sample. Findings suggest differentiation between positive and negative affective states, as well as between depressive and anxiety‐related features. These results support the potential utility of the item set for monitoring transient mood states in non‐clinical populations.

## Graphical abstract

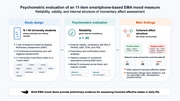

## 1 Introduction

The ubiquitous presence of mobile devices enables us to capture transitory emotional states in specific situations, which are often prone to recall inaccuracies. This technology allows us to understand how our daily activities and contexts dynamically influence our mental state. The potential is immense, particularly in envisioning ways to offer support precisely when it is most crucial (Schick et al. 2023). Smartphone‐based Ecological Momentary Assessments (EMA) are a feasible and efficient approach to capture emotional states and activities in people's everyday lives (Doherty et al. 2020), thereby reducing retrospective recall bias and enabling the study of temporal variability and associations (Myin‐Germeys et al. 2018).

Despite the increasing use of EMA, surprisingly little is known regarding its psychometric properties, and validated EMA measures remain scarce for several constructs, highlighting the need for EMA‐specific psychometric evaluation (Stone et al. 2020; Wrzus and Neubauer 2023; Murray et al. 2022; Hansen et al. 2025; Kleiman et al. 2023). To minimize participant burden, EMA studies often rely on very brief item sets; however, the impact of such brevity on reliability is seldom tested and therefore warrants explicit psychometric evaluation (Murray et al. 2022). Recent EMA validation work provides useful frameworks for evaluating reliability and construct validity in intensive longitudinal designs (Spangenberg et al. 2025; Forkmann et al. 2018). This is particularly important, as digital tools used to assess mood in real‐world settings are expected to meet demanding criteria: they should reliably capture short‐term fluctuations in affect, be robust to individual differences, including variations in mood and subclinical symptoms, and integrate seamlessly into everyday life (Stone et al. 2020; Flake and Fried 2020; Horstmann 2021).

Thus, the present study aimed to systematically investigate the psychometric properties of an 11‐item EMA of mood. Using EMA data collected over 1 week from a stress‐enriched non‐clinical student sample, we examined (1) internal consistency, (2) external validity with established retrospective psychometric scales, and (3) the factorial and network structure of the EMA to evaluate its suitability for digital mental health research and interventions. We expected the EMA to capture distinct affective dimensions and to show meaningful, though moderate, associations with established psychometric instruments.

## 2 Methods

### 2.1 Sample

EMA data from a randomized controlled trial investigating the effects of a 1‐week digital self‐efficacy training in a stress‐enriched non‐clinical student sample were used (Rohde et al. 2024). The inclusion criteria were being a student at a Swiss university, aged 18–29 years, owning a smartphone, speaking German fluently, and feeling stressed (i.e., a score of ≥13 on the Perceived Stress Scale; PSS; Cohen et al. 1983). Interested individuals with a self‐reported current or past psychiatric disorder were excluded; psychiatric disorders were not verified through a clinical interview. Data from a total of 93 participants were included. All participants provided informed consent electronically.

### 2.2 Study Design

Participants underwent a baseline assessment consisting of validated psychometric rating scales that had to be completed within 1 week. Thereafter, EMA was administered for seven days through SEMA3, an open‐source software application for smartphone surveys (O'Brien et al. 2024), which participants were asked to download via a link. All data were collected digitally. Following the seven‐day EMA period, participants completed a follow‐up assessment consisting of an extended set of validated psychometric rating scales that had to be completed within 1 week.

### 2.3 Questionnaires

#### 2.3.1 Perceived Stress Scale (PSS‐10)

The PSS is a self‐report measure assessing the degree to which individuals perceive situations as stressful (i.e., unpredictable, uncontrollable, or feeling overloaded). The scale consists of ten items, rated on a five‐point Likert scale, ranging from “never” (“0”) to “very often” (“4”). The sum score ranges from 0 to 40, with higher scores indicating greater levels of perceived stress (Cohen et al. 1983; Cohen 1988). Following commonly used interpretive guidelines, scores of 0–13 indicated low, 14–26 moderate, and 27–40 high perceived stress (Cohen et al. 1983).

#### 2.3.2 General Self‐Efficacy Scale (GSE)

The GSE is a self‐report questionnaire designed to measure an individual's belief in their ability to handle daily challenges. The scale comprises ten items, each rated on a four‐point Likert scale from “not at all true” (1) to “exactly true” (4). The sum score ranges from 10 to 40, where higher scores indicate stronger general self‐efficacy beliefs, and scores are typically interpreted continuously or descriptively, as normative data exist but no clinically validated cut‐off values are available (Tipton and Worthington 1984; Luszczynska et al. 2005).

#### 2.3.3 Positive and Negative Affect Schedule (PANAS)

The PANAS is a self‐report questionnaire measuring distinct dimensions of mood: positive affect (i.e., feelings such as alertness, enthusiasm, and joy) and negative affect (i.e., emotions like fear, sadness, and anger) (Watson et al. 1988). This results in the positive affect subscale (PANAS‐PA) and the negative affect subscale (PANAS‐NA). It consists of two 10‐item subscales, one for positive affect and one for negative affect. Respondents rate their momentary feelings on a five‐point Likert scale ranging from “not at all” (1) to “extremely” (5). Subscores range from 10 to 50, with higher scores representing higher positive or negative affect levels, respectively (Allan et al. 2015; Torres et al. 2023). Scores were interpreted continuously, as no well‐established clinical cut‐off values exist, with normative data from student samples indicating mean scores of approximately 29.7 (*SD* = 7.9) for PANAS‐PA and 14.8 (*SD* = 5.4) for PANAS‐NA (Watson et al. 1988).

#### 2.3.4 Beck Depression Inventory II (BDI‐II)

The BDI‐II is a self‐report questionnaire designed to assess the severity of depressive symptoms. It consists of 21 items. Respondents rate each statement based on how they have felt over the past 2 weeks on a four‐point Likert scale from “absent” (0) to “severe” (3). The BDI‐II sum score ranges from 0 to 63 (Beck 1996). Following established guidelines, total scores of 0–13 indicate minimal, 14–19 mild, 20–28 moderate, and 29–63 severe depressive symptoms (Beck 1996). Since its introduction, the BDI‐II has become a valuable tool for screening and monitoring depression in clinical and research settings (Beck et al. 1988; Wang and Gorenstein 2013).

#### 2.3.5 State and Trait Anxiety Inventory (STAI)

The STAI is a self‐report instrument that assesses two distinct but related dimensions of anxiety: state (i.e., current, temporary) anxiety and trait (i.e., the propensity to experience) anxiety. This results in the trait anxiety subscale (STAI‐T) and the state anxiety subscale (STAI‐S). The STAI consists of two 20‐item subscales ranging from 20 to 80. Respondents rate each item on a four‐point Likert scale, ranging from “not at all” (1) to “very much” (4) (Skapinakis 2014; Spielberger et al. 1983). Total scores are interpreted continuously, with commonly used cut‐off guidelines indicating low (20–37), moderate (38–44), and high (45–80) anxiety levels for both subscales (Spielberger et al. 1970; Knight et al. 1983).

### 2.4 Ecological Momentary Assessment

Momentary mood was assessed using an 11‐item smartphone‐based EMA set, which was first developed in a clinical sample (Vaessen et al. 2019) and then adapted for the non‐clinical context (Marciniak et al. 2024), but has not been validated so far. It has subsequently been used in multiple studies by our group and others (Bögemann et al. 2023; Marciniak et al. 2023; Marciniak et al. 2024; Wackerhagen et al. 2023; Rohde et al. 2026). In this study, EMA items were administered in German; Table 1 reports the original German wording alongside an English translation for readability.

**TABLE 1:** Overview of the Ecological Momentary Assessment mood item set.

No.	Item label	Item wording (German)	English translation
1	Cheerful	Ich bin heiter.	I feel cheerful.
2	Irritated	Ich fühle mich gereizt.	I feel irritated.
3	Anxious	Ich fühle mich besorgt.	I feel anxious.
4	Happy	Ich fühle mich zufrieden.	I feel happy.
5	Insecure	Ich fühle mich unsicher.	I feel insecure.
6	Lonely	Ich fühle mich einsam.	I feel lonely.
7	Relaxed	Ich fühle mich entspannt.	I feel relaxed.
8	Sad	Ich fühle mich traurig.	I feel sad.
9	Overthinking	Es beschäftigen mich immer die gleichen Gedanken.	I cannot stop overthinking things.
10	Focused	Ich kann mich gerade gut konzentrieren.	I can concentrate well.
11	Stressed	Ich fühle mich gestresst.	I feel stressed.

*Note*: In the study, we used the German version of the EMA items.

Participants were asked to rate each of the items using a 7‐point slider scale ranging from “not at all” (1) to “very much” (7). EMA was administered for 1 week via the SEMA3 smartphone application, which provides advanced smartphone surveys (O'Brien et al. 2024). Participants were prompted 10 times per day at fixed 30‐min intervals between 8:30 AM and 10:30 PM. This sampling density is commonly used to capture highly time‐varying constructs such as mood while balancing participant burden (Myin‐Germeys et al. 2018). They were asked to respond within 20 min after standard prompts; in prompts that were followed by the digital self‐efficacy training module, the response window was extended to 50 min to accommodate the completion of the module. Additionally, participants were able to self‐trigger EMA. Each EMA assessment consisted of the same 11 mood items. Completion was defined as answering all 11 items. For reporting feasibility and missingness, we distinguished between assessment‐level counts, that is, scheduled and self‐triggered EMA assessments, and item‐level responses.

The target construct of the EMA instrument is momentary affective state, defined as the participant's subjective emotional experience at the time of each assessment prompt. Each EMA item is intended to capture a distinct affective state (e.g., sadness, anger, happiness) as a state‐like, time‐varying experience, rather than a stable trait. Accordingly, the primary unit of interpretation is the single‐item rating at each EMA assessment, which represents the intensity of the corresponding affective state at that specific moment. No composite score was assumed a priori at the assessment level, and individual items were analyzed as separate indicators of momentary mood states.

### 2.5 Statistical Analysis

We used descriptive statistics (mean, *M*, standard deviation, *SD*, and percentages) to represent the demographic and psychological characteristics of our sample. The analysis of primary and secondary outcomes, as well as how EMA parameters interacted with each other and with baseline variables, has been published previously (Rohde et al. 2024; Rohde et al. 2023).

EMA observations were treated as the unit of analysis to characterize the overall covariance structure of the 11‐item mood measure across all assessments. This single‐level pooling does not separate within‐person from between‐person variance; therefore, results are interpreted descriptively, reflecting a mixture of state‐ and trait‐like associations rather than purely inferential conclusions. Item distributions were examined using skewness and kurtosis, and internal consistency was assessed with Cronbach's alpha (*α*).

Given the state‐based nature of the target construct, psychometric analyses primarily focused on assessment‐level EMA observations, treating each item response as an indicator of momentary affective state. Analyses of internal structure (factorial and network models) therefore reflect the covariance structure among momentary state indicators across assessments, without separating within‐ and between‐person variance. These results are interpreted descriptively as evidence regarding the coherence and organization of affective states as captured by the EMA items.

For external validity, EMA responses were aggregated at the participant level (mean of each item across assessments within the first 24 h of app use) and correlated with baseline psychometric questionnaires using Pearson's *r*. For interpretation of correlation strength, absolute values were considered, while the direction of associations was interpreted based on the signed correlation coefficients. A correlation between 0.00 and 0.39 was considered weak, a correlation between 0.40 and 0.69 was considered moderate, and a correlation between 0.70 and 1.00 was considered strong (Schober et al. 2018). A post hoc power analysis indicated that a minimum of 62 participants would have been sufficient to detect moderate correlations, suggesting that the available sample size (*n* = 93) provided adequate power. Concordance and agreement with baseline measures were further evaluated using concordance correlation coefficients and a Bland–Altman plot.

Factorial analyses were conducted on the EMA assessments outside the first 24 h of app use to minimize confirmation bias, with the aim of exploring the latent structure underlying the set of momentary mood items rather than establishing definitive subscales. The assessments were randomly split into two subsamples for exploratory factor analysis (EFA) and confirmatory factor analysis (CFA). EFA assumptions were evaluated using the Kaiser–Meyer–Olkin (KMO) measure and Bartlett's test of sphericity, and eigenvalues were visualized with a scree plot. To determine the appropriate rotation method (oblique vs. orthogonal), a sensitivity analysis was conducted examining factor correlations, with values exceeding 0.30 indicating that an oblique rotation was more appropriate. The EFA was used to identify candidate dimensions reflecting patterns of covariation among items across repeated momentary assessments. CFA was subsequently applied to evaluate the adequacy of these candidate structures in a holdout set of assessments, serving as a structural consistency check rather than as a formal scale validation procedure.

In addition, network analysis was conducted to provide a complementary, non‐latent‐variable‐based perspective on the internal organization of the EMA items. Factor and network approaches were interpreted as complementary analytic frameworks addressing different aspects of the EMA item structure rather than as directly competing models. A Gaussian graphical model with LASSO regularization and EBIC selection (*γ* = 0.0) was estimated to identify robust conditional associations among items, considering the number of participants favoring accuracy over sparsity. In this framework, edges represent regularized partial correlations, and network communities and centrality indices (strength, closeness, betweenness, and expected influence) were examined to characterize the relative interconnectedness and potential functional roles of individual items (Borsboom et al. 2021). Importantly, the identified communities are interpreted as heuristic groupings that reflect empirical association patterns and conditional dependencies among momentary ratings rather than validated subscales or discrete psychological constructs. Network stability and edge‐weight accuracy were evaluated using non‐parametric bootstrapping (10’000 resamples).

CFA model fit was assessed with multiple indices, including chi‐square (*χ* ^2^), Comparative Fit Index (CFI), Tucker‐Lewis Index (TLI), Akaike Information Criterion (AIC), Bayesian Information Criterion (BIC), and Root Mean Square Error of Approximation (RMSEA). chi‐square compares the discrepancy between observed and model‐implied covariances, with smaller values indicating a better fit. The CFI and TLI compare model fit to a null (independence) model, with values close to 1.00 (typically ≥ 0.95), indicating a good fit. The AIC and BIC balance model fit and complexity, with BIC penalizing complexity more strongly to favor simpler models. The RMSEA measures the discrepancy between the hypothesized model and the data per degree of freedom, with values ≤ 0.06 generally considered indicative of a good fit. Since some network centrality measures are roughly equivalent to factor loadings, these parameters allow for comparison between psychometric factor and network models (Kan et al. 2020; Christensen and Golino 2021).

For statistical analyses and figures, we used RStudio (2026.05.0+218), the statistical software R (version 4.6.0), using the R packages tidyverse (version 2.0.0), fmsb (version 0.7.6), rstatix (version 0.7.2), factoextra (version 1.0.7), FactoMineR (version 2.11), GPArotation (version 2024.3‐1), psych (version 2.4.3), lavaan (version 0.6‐18), qgraph (version 1.9.8), psychometrics (version 0.12), bootnet (version 1.6), and networktools (version 1.5.2) (Borsboom et al. 2021).

## 3 Results

A total of 93 students with a mean age of 23.27 (*SD* = 3.49) years participated in the study. Table 2 summarizes basic demographic and psychological characteristics. At the assessment level, 6509 scheduled EMA prompts were generated, of which 3538 were completed (completion rate 54.4%). In addition, 1232 self‐triggered EMA assessments were initiated, of which 1118 were completed (completion rate 90.7%). At the item level, participants provided 53,578 completed item responses out of 85,151 possible item‐level response opportunities, corresponding to an item‐level response rate of 62.9%. Specifically, scheduled prompts accounted for 71,599 item‐level response opportunities, of which 40,463 were completed (56.5%), and self‐triggered assessments accounted for 13,552 item‐level response opportunities, of which 13,115 were completed (96.8%). For an overview of the proportion of completed item responses across EMA assessment periods by item, see Figure S1.

**TABLE 2:** Demographic and baseline characteristics of the sample.

Demographic characteristics	Mean (*SD*)
Age (in years)	23.27 (3.49)
Sex	***n* (%)**
Male	20 (22)
Female	73 (79)
Education	
Regular education/apprenticeship	46 (49)
Bachelor	38 (41)
Master	9 (10)
Civil status	
Single	36 (39)
Relationship	41 (44)
Married/living together	16 (17)
Questionnaires	**Mean (*SD*)**
BDI‐II	12.99 (9.02)
STAI trait	43.84 (10.74)
STAI state	43.13 (11.18)
PANAS positive	20.81 (4.75)
PANAS negative	22.69 (4.48)
GSE	27.34 (4.36)
PSS	21.98 (5.93)

BDI‐II, Beck Depression Inventory II; PANAS, Positive and Negative Affect Schedule; STAI, State and Trait Anxiety Inventory; GSE, General Self‐Efficacy Scale; PSS, Perceived Stress Scale.

### 3.1 Demographic Characteristics of the Sample

**FIGURE 1:**
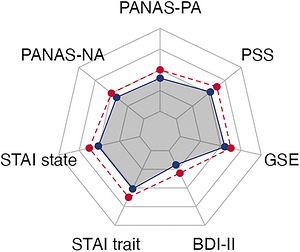
Psychometric characteristics of the sample. BDI‐II, Beck Depression Inventory‐II; PANAS, Positive and Negative Affect Schedule; STAI, State and Trait Anxiety Inventory; GSE, General Self‐Efficacy Scale; PSS, Perceived Stress Scale. *Note*: The blue line represents the sample characteristics. The red line represents commonly used reference values/cut‐points where available.

Participants were mostly female (79%, *n* = 73; Table 2). All were university students with half having completed either a bachelor's or master's degree (51%, *n* = 47). Most participants were in a stable relationship (61%, *n* = 57). The sample showed minimally depressive symptoms (BDI‐II: *M* = 12.99, *SD* = 9.02) and moderately anxiety levels (STAI‐S: *M* = 43.13, *SD* = 11.18; STAI‐T: *M* = 43.84, *SD* = 10.74). Mean positive affect (PANAS‐PA) was 20.81 (*SD* = 4.75) and mean negative affect (PANAS‐NA) was 22.69 (*SD* = 4.48). The mean general self‐efficacy score (GSE) was 27.34 (*SD* = 4.36). Finally, perceived stress (PSS) averaged 21.98 (*SD* = 5.93). Figure 1 represents the psychometric profile of the sample using commonly used reference values or cut‐points where available.

### 3.2 Variability, Reliability, and Validity of EMA Items

We examined descriptive statistics, measures of variability, and reliability estimates for the EMA item set (Table 3 and Figure S2). All mean squared successive differences (MSSDs) were above zero, indicating that participants exhibited within‐person fluctuations over time. At the single‐item level, more items assessing positive affect (i.e., cheerful, relaxed, focused) than negative affect (i.e., anxious, stressed) showed the highest mean MSSDs, reflecting pronounced point‐to‐point variability. In contrast, items related to insecurity, loneliness, and sadness demonstrated the lowest mean MSSDs, suggesting more stable responses across time. Despite these differences in average variability, the range of individual MSSDs was considerable for most items, highlighting substantial inter‐individual differences in moment‐to‐moment variability. Intraclass correlations (ICCs) indicated that between‐person variability was generally higher (i.e., between 0.48 and 0.57) for items with lower mean MSSDs but broader individual ranges (e.g., insecure, lonely, and sad), suggesting that these items capture more trait‐like differences across participants. Overall, these results indicate that the EMA items varied in both their temporal dynamics and reliability, with some items showing stronger moment‐to‐moment fluctuations and others reflecting more stable, between‐person differences.

**TABLE 3:** Descriptive statistics, variability indices, and reliability of the EMA items and scales.

Variable	*M*	*SD*	Min	Max	MSSD (*M*)	MSSD (*SD*)	Min MSSD	Max MSSD	ICC	Rel (person)	Rel (moment)
Cheerful	4.54	1.51	1	7	2.02	1.43	0.16	7.32	0.36	0.36	0.64
Irritated	2.26	1.57	1	7	1.94	1.61	0.00	8.78	0.49	0.49	0.51
Anxious	2.67	1.71	1	7	2.11	1.68	0.00	8.71	0.51	0.51	0.49
Happy	4.87	1.48	1	7	1.83	1.15	0.16	5.57	0.35	0.35	0.65
Insecure	2.46	1.66	1	7	1.69	1.55	0.00	9.07	0.57	0.57	0.43
Lonely	2.02	1.45	1	7	1.60	1.48	0.00	6.50	0.48	0.48	0.52
Relaxed	4.42	1.56	1	7	2.53	1.29	0.05	6.10	0.25	0.25	0.75
Sad	2.04	1.49	1	7	1.43	1.27	0.00	5.09	0.51	0.51	0.49
Overthinking	3.13	1.87	1	7	1.74	1.46	0.04	7.21	0.61	0.61	0.39
Focused	4.16	1.53	1	7	2.74	1.68	0.61	12.19	0.24	0.24	0.76
Stressed	3.06	1.87	1	7	2.24	1.51	0.00	9.15	0.46	0.46	0.54

*Note*: Reliability estimates follow variance decomposition approaches commonly used in EMA research.

MSSD, mean squared successive differences; ICC, intraclass correlation coefficient from random‐intercept models.

### 3.3 External Validation of the EMA

For the external validity analysis, we included all EMAs completed until the end of the first day of app participation (817 completed EMAs). The EMA items showed a normal distribution, with skewness values between –0.49 and 1.43 and kurtosis values between –0.60 and 1.11. The internal consistency was good, with a Cronbach's *α* of 0.88. We correlated individual EMA items with established psychometric scales (Table 4).

**TABLE 4:** Correlation coefficients of the single EMA items with the questionnaires.

	BDI‐II	PANAS positive	PANAS negative	STAI trait	STAI state	GSE	PSS
Items	Correlation (95% CI)	Correlation (95% CI)	Correlation (95% CI)	Correlation (95% CI)	Correlation (95% CI)	Correlation (95% CI)	Correlation (95% CI)
Relaxed	−0.19 (–0.26 to –0.12)	−0.08 (–0.16 to –0.01)	0.17 (0.10 to 0.24)	−0.20 (–0.27 to –0.13)	−0.24 (–0.30 to –0.17)	0.13 (0.07 to 0.21)	−0.09 (–0.16 to –0.02)
Cheerful	−0.19 (–0.26 to –0.11)	0.24 (0.15 to 0.33)	0.18 (0.11 to 0.25)	−0.25 (–0.32 to –0.18)	−0.24 (–0.31 to –0.17)	0.21 (0.13 to 0.28)	−0.08 (–0.16 to –0.01)
Happy	−0.27 (–0.34 to –0.20)	0.26 (0.18 to 0.32)	0.21 (0.14 to 0.29)	−0.30 (–0.36 to –0.23)	−0.25 (–0.32 to –0.18)	0.26 (0.18 to 0.32)	−0.17 (–0.24 to –0.09)
Focused	−0.15 (–0.22 to –0.07)	0.11 (0.03 to 0.19)	0.16 (0.09 to 0.23)	−0.20 (–0.27 to –0.13)	−0.19 (–0.26 to –0.12)	0.17 (0.10 to 0.25)	−0.04 (–0.11 to –0.03)
Insecure	0.23 (0.17 to 0.31)	0.08 (0.01 to 0.15)	0.42 (0.36 to 0.49)	0.47 (0.41 to 0.53)	0.39 (0.33 to 0.45)	−0.37 (–0.43 to –0.30)	0.34 (0.28 to 0.41)
Anxious	0.29 (0.22 to 0.36)	0.07 (–0.01 to 0.15)	0.45 (0.39 to 0.51)	0.48 (0.42 to 0.53)	0.40 (0.33 to 0.46)	−0.34 (–0.41 to –0.28)	0.40 (0.34 to 0.46)
Over‐thinking	0.23 (0.16 to 0.30)	−0.02 (–0.09 to 0.06)	0.37 (0.31 to 0.44)	0.45 (0.39 to 0.51)	0.37 (0.30 to 0.43)	−0.39 (–0.45 to –0.33)	0.15 (0.07 to 0.22)
Stressed	0.17 (0.09 to 0.24)	0.02 (–0.06 to 0.08)	0.30 (0.24 to 0.38)	0.36 (0.30 to 0.43)	0.33 (0.26 to 0.40)	−0.20 (–0.28 to –0.13)	0.23 (0.16 to 0.31)
Irritated	0.22 (0.15 to 0.29)	0.10 (0.03 to 0.18)	0.42 (0.35 to 0.48)	0.31 (0.24 to 0.38)	0.32 (0.24 to 0.38)	−0.23 (–0.30 to –0.16)	0.34 (0.28 to 0.41)
Sad	0.25 (0.18 to 0.32)	0.04 (–0.03 to 0.12)	0.41 (0.35 to 0.47)	0.45 (0.39 to 0.51)	0.34 (0.28 to 0.41)	−0.31 (–0.38 to –0.25)	0.31 (0.24 to 0.38)
Lonely	0.11 (0.04 to 0.19)	0.08 (0.01 to 0.16)	0.27 (0.20 to 0.34)	0.31 (0.25 to 0.38)	0.24 (0.17 to 0.31)	−0.23 (–0.31 to –0.16)	0.21 (0.14 to 0.28)

*Note*. Values are Pearson's *r* with 95% confidence intervals. Correlation strength was interpreted based on absolute values: |*r*| = 0.00–0.39 = low, |*r*| = 0.40–0.69 = moderate, and |*r*| = 0.70–1.00 = strong.

BDI‐II, Beck Depression Inventory II; PANAS, Positive and Negative Affect Schedule; STAI, State and Trait Anxiety Inventory; GSE, General Self‐Efficacy Scale; PSS, Perceived Stress Scale.

Positively valenced EMA items (“relaxed,” “cheerful,” “happy,” and “focused”) were negatively associated with depression severity (BDI‐II; *r* = –0.15 to –0.27), trait anxiety (STAI‐T; *r* = –0.20 to –0.30), and state anxiety (STAI‐S; *r* = –0.19 to –0.25), while showing positive associations with general self‐efficacy (GSE; *r* = 0.13 to 0.26). Associations with perceived stress (PSS) were small and negative (*r* = –0.04 to –0.17).

In contrast, negatively valenced EMA items (“insecure,” “anxious,” “irritated,” “sad,” and “lonely”) showed positive correlations with depression severity (*r* = 0.11 to 0.29), trait anxiety (STAI‐T; *r* = 0.31 to 0.48), state anxiety (STAI‐S; *r* = 0.24 to 0.40), and perceived stress (PSS; *r* = 0.15 to 0.40), while being negatively associated with self‐efficacy (GSE; *r* = –0.20 to –0.39). The strongest associations were observed between “anxious” and negative affect (PANAS‐NA; *r* = 0.45), trait anxiety (*r* = 0.48), and perceived stress (*r* = 0.40), as well as between “insecure” and trait anxiety (*r* = 0.47).

Overall, the pattern of correlations generally supported the convergent validity of the EMA items, with positively valenced EMA states relating to lower psychopathology and higher self‐efficacy, and negatively valenced EMA states relating to greater emotional distress, anxiety, and stress. Concordance correlation coefficients between the aggregated EMA mood score and the psychometric instruments were low (range: 0.05–0.15), indicating limited concordance between momentary EMA assessments and retrospective questionnaire measures. Bland–Altman analyses showed no substantial systematic bias, with only one observation outside the limits of agreement for PANAS‐NA, PSS, and GSE, and two observations outside the limits for the BDI‐II (Table S1). Because the EMA item set was designed to capture momentary affective states rather than global retrospective evaluations, associations with established psychometric instruments were interpreted primarily at the level of individual EMA items; analyses using aggregated EMA mood scores were considered supplementary and descriptive.

### 3.4 Internal Validation of the EMA

#### 3.4.1 Exploratory Factor Analysis (EFA)

We used the remaining EMAs (i.e., all EMA assessments outside the first 24 h of app use; 3839 completed EMAs) for the factor analysis and randomly created two subsamples, one for the EFA and one for the CFA. The KMO measure verified the sampling adequacy for further analysis (KMO = 0.91), and all KMO values for the single items were > 0.87, which is above the minimum limit of 0.50. In the EFA subsample, Bartlett's test of sphericity indicated that the correlations between the items were sufficient for analysis (*χ* ^2^(55) = 10’651.6, *p* < 0.001). Two factors had eigenvalues over Kaiser's criterion of 1. The scree plot was ambiguous and showed an inflection that could justify retaining three factors. Examination of factor correlations in the sensitivity analysis showed values exceeding .50, indicating that an oblique rotation was more appropriate than an orthogonal rotation. Therefore, an exploratory factor analysis using oblimin rotation was conducted on the 11 EMA items. Considering the large sample size and the ambiguous results of the scree plot, we decided to analyze both a two‐ and three‐factor solution (see Table 5).

**TABLE 5:** Results from the Exploratory Factorial Analysis of the EMA (oblimin rotation).

	One factor	Two factor		Three factor		
	Factor 01	Factor 01	Factor 02	Factor 01	Factor 02	Factor 03
		Positive mood	Negative mood	Positive mood	Negative mood (anxious/ distressed)	Negative mood (depressive)
Relaxed	−0.67	**0.82**	−0.01	**0.75**	−0.21	−0.17
Cheerful	−0.67	**0.80**	−0.03	**0.83**	0.13	−0.22
Happy	−0.75	**0.79**	−0.12	**0.80**	−0.03	−0.15
Focused	−0.43	**0.71**	0.15	**0.64**	−0.09	0.23
Insecure	0.78	0.04	**0.87**	0.08	**0.85**	0.12
Anxious	0.81	−0.03	**0.83**	0.01	**0.84**	0.10
Overthinking	0.70	0.02	**0.76**	0.04	**0.72**	0.13
Stressed	0.76	−0.28	**0.56**	−0.19	**0.80**	−0.17
Irritated	0.72	−0.23	**0.56**	−0.23	**0.47**	0.18
Sad	0.77	−0.04	**0.79**	−0.15	0.26	**0.67**
Lonely	0.65	0.10	**0.78**	−0.06	0.10	**0.82**

*Note*: Values are standardized factor loadings from exploratory factor analyses of 11 EMA affect items.

Factor labels reflect item content: Positive Mood (Relaxed, Cheerful, Happy, Focused), Negative Mood—anxious/distressed (Insecure, Anxious, Overthinking, Stressed, Irritated), and Negative Mood—depressive (Sad, Lonely).

Loadings > |0.45| are highlighted in bold for interpretation.

Some items show cross‐loadings, especially in the three‐factor solution, indicating overlap between negative affect dimensions.

Oblimin rotation was applied; higher absolute loadings indicate stronger associations with the factor.

#### 3.4.2 Network Analysis of the EMA

**FIGURE 2:**
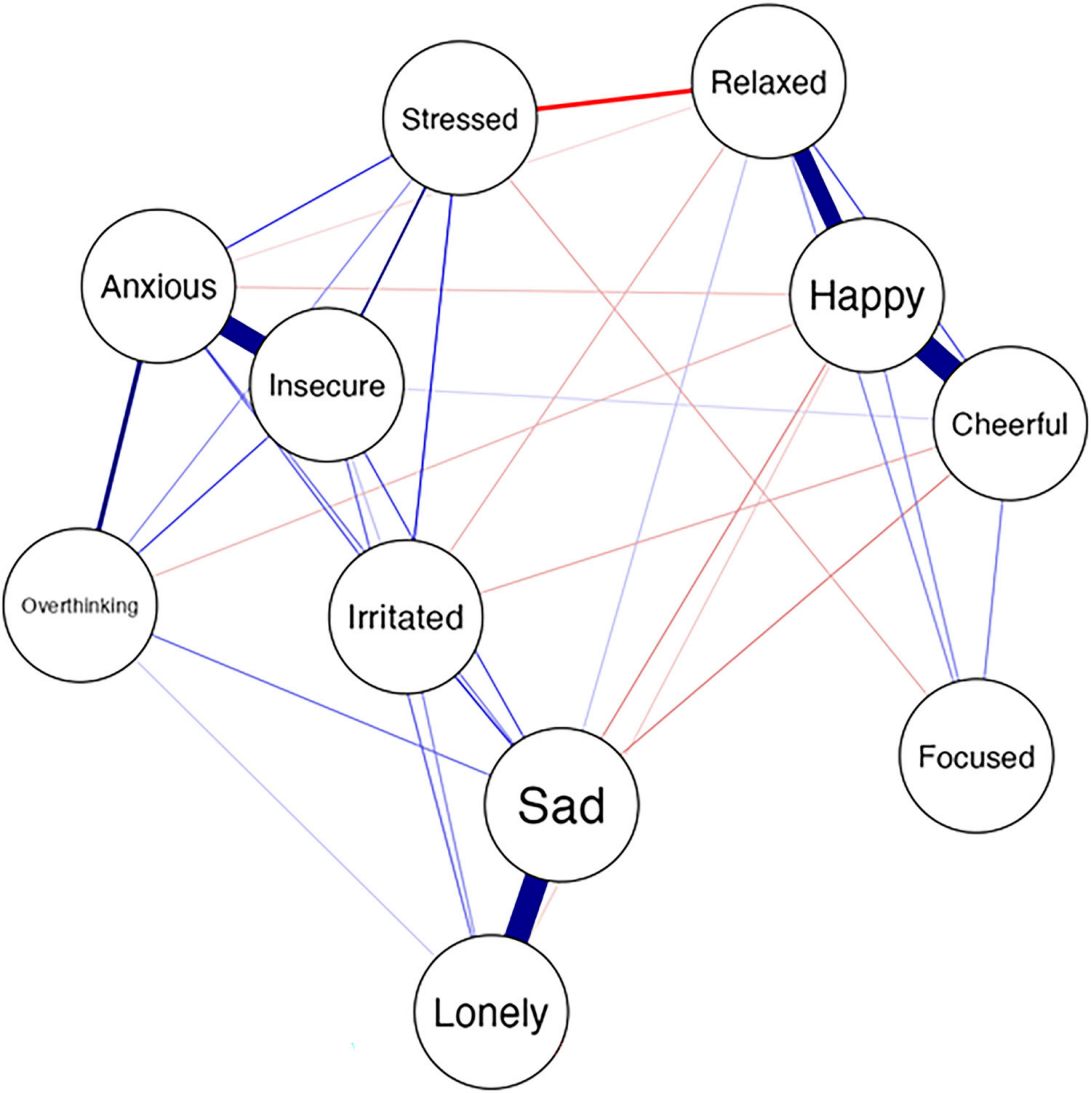
EMA affect network and centrality. *Note*: Network of momentary affective states estimated from Ecological Momentary Assessment (EMA) data using a regularized partial correlation model. Nodes represent affect items, and edges represent conditional associations after controlling for all other nodes. Edge thickness indicates association strength; blue edges indicate positive associations, and red edges indicate negative associations. The network layout was generated using the Fruchterman–Reingold algorithm, positioning more strongly connected nodes closer together. Two communities corresponding to positive and negative affective states are visible. Network stability was excellent (correlation stability coefficient = 0.89). Centrality indices (strength, closeness, betweenness, and expected influence) were computed to describe the relative importance of nodes within the network.

The EMA network (Figure 2) exhibited excellent stability, with a correlation stability coefficient of 0.89. Two well‐defined communities emerged, corresponding to positive and negative affective states. Nodes showed a mean strength of 0.80 (*SD* = 0.14), with node 08 (“sad”) having the highest strength, suggesting strong connectivity with other affective states. Mean node closeness was 0.89 (*SD* = 0.07), again with “sad” showing the highest value, indicating a central position within the network. Betweenness centrality was moderate overall (*M* = 0.49, *SD* = 0.24), with node 04 (“happy”) displaying the highest betweenness, indicating a relatively central position in the observed network structure. Finally, expected influence averaged 0.42 (*SD* = 0.24), with node 05 (“insecure”) showing the highest expected influence, reflecting the signed pattern of its associations with other emotional states (see Figure 2).

Centrality analyses indicated that “sad” was the most central node in terms of strength and closeness, “happy” showed the highest betweenness, and “insecure” exhibited the highest expected influence, suggesting their relative importance within the EMA affect network (see Figure S3 for full centrality plots).

#### 3.4.3 Confirmatory Analysis of the Factorial and Network Models of the EMA

The *χ* ^2^ tests were significant for all models, reflecting statistically detectable discrepancies between observed and model‐implied covariance structures. Given the sensitivity of *χ* ^2^ to sample size, model fit was primarily interpreted using CFI, TLI, AIC, BIC, and RMSEA. Only the three‐factor and network model achieved CFI ≥ 0.95, while only the network model met the TLI criterion. AIC and BIC were lowest for the network model, indicating the most favorable balance between fit and parsimony among the models examined. Finally, RMSEA indicated good fit for the network model (≤ 0.06), with the three‐factor model approaching this threshold. In summary, the network model showed the most favorable fit indices among the models examined (Table S2).

#### 3.4.4 Integration of Factorial and Network Analyses

The EMA affect network revealed a coherent structure with two communities corresponding to positive and negative affective states. Network stability was excellent (correlation stability coefficient = 0.89), and edges represented regularized partial correlations between items (Figure 2). Centrality analyses indicated that “sad” was the most central node in terms of strength and closeness, “happy” exhibited the highest betweenness, and “insecure” showed the highest expected influence, suggesting their relative importance within the network (see Figure S3). Complementary factorial analyses supported a partially overlapping interpretation: the network model showed the most favorable fit indices among the examined models (CFI = 0.99, RMSEA = 0.01), while the three‐factor solution also showed acceptable fit (see Table S2). Together, these findings indicate that the EMA items capture coherent affective states that are empirically grouped and differentially connected within the observed affect network.

## 4 Discussion

We examined the psychometric properties and structural organization of a brief 11‐item smartphone‐based EMA mood scale in 93 university students who completed 1 week of intensive assessments, averaging seven prompts per day. The EMA items showed good overall internal consistency (Cronbach's alpha = 0.88), although traditional reliability indices must be interpreted cautiously in intensive longitudinal designs. Convergent validity with retrospective self‐report measures was modest, with moderate correlations observed primarily with the PANAS (PANAS‐NA) negative affect subscale, STAI trait (STAI‐T), STAI state (STAI‐S), and perceived stress (PSS), suggesting that the EMA items capture momentary affective states that are related to, but not redundant with, trait‐like constructs. Exploratory factor analysis yielded ambiguous results, supporting either a two‐ or three‐factor solution, despite excellent sampling adequacy (KMO = 0.91). In contrast, network analysis revealed a stable, coherent affective structure consisting of two well‐defined communities corresponding to positive and negative affective states. Confirmatory model comparison further indicated that the network model showed the most favorable fit indices among the models examined (CFI, TLI, AIC, BIC, and RMSEA), supporting its usefulness as a complementary framework for describing conditional associations among EMA mood items.

We investigated a stress‐enriched non‐clinical student sample, as indicated by baseline scores that were largely below clinically relevant thresholds. For instance, mean depression severity measured using the BDI‐II was 12.99 (*SD* = 9.02), which is below the cutoff for moderate depression (Beck, 1996). Various psychological constructs, such as affect states, anxiety, and stress, were assessed using established self‐report instruments. The measures are not intended for clinical diagnosis but rather to capture psychological well‐being and symptom‐related experiences across both clinical and non‐clinical populations (Cohen et al. 1983; Beck et al. 1988; Skapinakis 2014; Crawford and Henry 2004).

The predominantly weak correlations between EMA items and retrospective questionnaires are consistent with the non‐clinical nature of the sample and the conceptual distinction between momentary affective experiences and aggregated trait‐like symptom reports. At the item level, EMA items related to distress or anxiety (e.g., “insecure,” “anxious,” “sad,” “irritated,” “overthinking”) showed moderate correlations with retrospective psychometric measures. These items correlated most strongly with the STAI trait and state subscales, as well as the PANAS negative affect scale. The EMA item “anxious” showed consistent associations across all three (Watson et al. 1988; Spielberger et al. 1983), while “sad” and “insecure” were also moderately related to perceived stress (PSS). In contrast, correlations with depressive symptoms (BDI‐II) and general self‐efficacy were mostly low, as anticipated given that EMA items capture short‐term affective fluctuations rather than the severity of depressive symptomatology. These findings align with prior research suggesting that EMA captures context‐sensitive and short‐term affective fluctuations that only partially overlap with trait‐based or diagnostic instruments (Conner and Barrett 2012; Stein and Nesse 2015). This is further supported by the finding that the EMA item “stressed” showed only a low correlation with the PSS, which reflects perceived stress over the past month (Cohen et al. 1983). In contrast, the item “anxious” correlated moderately with both STAI‐State and PSS, consistent with studies showing that visual analog scales may be particularly sensitive to short‐term anxiety (Williams et al. 2010). Together, these results provide preliminary support for the convergent validity of EMA in assessing transient affective states, especially anxiety‐related experiences, while also underscoring its conceptual distinction from traditional retrospective self‐report instruments.

### 4.1 Internal Structure: Factorial and Network Perspective

Our findings from the exploratory factor analyses provided evidence for a coherent but not unequivocal latent structure of the EMA mood items. Sampling adequacy was excellent (i.e., high KMO value and consistently strong item‐level KMO indices), confirming that the data were well suited for factorial analysis. Overall, the items appeared to cluster into two broad affective dimensions, but the structure was not sharply defined, leaving room for a more differentiated representation. We therefore considered both simpler and more complex solutions in follow‐up analyses. This ambiguity suggests that while the EMA items reflect underlying affective dimensions, their momentary nature may limit the emergence of sharply differentiated latent factors, supporting the notion that affective experiences assessed in real time are multidimensional and partially overlapping rather than cleanly separable into distinct latent constructs.

Despite the modest convergence with retrospective self‐rating measures, the network model provided complementary insights into the structure of the EMA mood item set. The estimated network exhibited high stability (CS = 0.89), supporting the robustness of the observed associations. However, these associations reflect pooled between‐ and within‐person variation and therefore should not be interpreted as evidence for person‐specific dynamic processes. The network revealed two well‐defined communities corresponding broadly to negative affective states (including “stressed,” “anxious,” “irritated,” “overthinking,” “sad,” “insecure,” and “lonely”) and positive affective states (including “relaxed,” “happy,” “cheerful,” and “focused”), consistent with prior EMA and affect network research (Hall et al. 2021). This structure largely overlapped with the two‐factor interpretation identified in the factor analyses. In contrast, the three‐factor solution suggested a potential differentiation within the negative affect domain; however, given the observed cross‐loadings and the comparative model‐fit pattern, this structure should be considered tentative and interpreted with caution. From both clinical and psychometric perspectives, distinguishing between different facets of negative affect remains challenging. Centrality analyses further described differences in the relative position of individual affective states within the network. The item “sad” showed the highest strength and closeness, indicating strong connectivity with other affective states and a central position within the network structure. In contrast, “happy” showed the highest betweenness centrality, and “insecure” showed the highest expected influence. These indices indicate that these items were relatively prominent within the observed conditional association structure, but should not be interpreted as evidence for causal importance or temporal influence.

Within the negative affect community, the item “overthinking” was closely connected to “anxious,” consistent with interpretations reflecting ruminative or insight‐related processes rather than externally oriented cognitive engagement (Dehghan Sarvolia and Dehghani 2019; Huddy et al. 2008; Dorjee 2016). In contrast, “focused” clustered with positive affective states and may reflect goal‐directed, externally oriented cognitive functioning (Legrenzi et al. 1993). Together, these findings suggest that momentary affective states assessed via EMA form an interconnected pattern of associations in which some emotions appear more strongly connected than others. However, given the pooled analytical approach, these findings should be interpreted as descriptive of the observed data structure rather than evidence for stable underlying affective mechanisms or causal relationships.

Our results reflect the relationships between transient emotions within the non‐pathological range of experience (Gross and Jazaieri 2014; Houben et al. 2015). Furthermore, the relation of the single EMA measurement to more stable and long‐lasting emotional states (as assessed with psychometric measures) was modest, highlighting that the primary strength of the EMA scale may lie in capturing momentary affective experiences and their interrelations rather than stable trait‐like characteristics. Exploratory and confirmatory structure analyses of the EMA revealed a potentially complex structure, with positive affect factors and negative affect factors. The latter showed possible differentiation into two subfactors: anxious/distressed and depressive. This distinction should be interpreted cautiously given the observed cross‐loadings and the inability of the analyses to disentangle within‐person from between‐person variance. The pivotal axis between the constructs appeared to involve stress‐relaxed, closely related to insecurity, indicating a possible role of confidence in coping with daily life. The network analysis further elucidated the relative position of individual affective states. Centrality metrics indicated that “sad” was the most central node in terms of strength and closeness, “happy” exhibited the highest betweenness, and “insecure” showed the highest expected influence, suggesting that these states were relatively prominent within the observed network structure. Centrality indices in cross‐sectional pooled networks should be interpreted cautiously and do not necessarily reflect causal importance or temporal influence at the individual level. These centrality patterns should therefore be interpreted as descriptive indicator of the observed EMA affect network rather than as indicators of symptom severity or temporal influence.

Interpreted in light of broader psychological frameworks, these findings underscore the importance of both negative and positive affect as general mood descriptors. Positive affect emphasizes resources rather than deficits, aligning with a salutogenic perspective (Hall et al. 2021). Differentiating negative mood into anxious/distressed and depressive aspects may allow for a more specific characterization of the negative affect profile, supported by literature showing that anxiety and sadness manifest and affect decision‐making differently (Losada‐Baltar et al. 2020; Raghunathan and Pham 1999). The observed associations involving “stressed,” “relaxed,” “focused,” and “overthinking” may contribute to the characterization of the conditional association structure of momentary affect (Houben et al. 2015). Together, these observations suggest that centrality indices can help describe the relative position and connectedness of individual EMA items within the observed affect network, without implying causal, regulatory, or temporal influence.

Taken together, the factorial and network analyses provide complementary perspectives on the structure of momentary affect assessed via EMA. While confirmatory factor analyses indicated that a three‐factor solution achieved acceptable fit, the network model showed comparatively favorable fit and information criteria relative to the factorial models across multiple fit indices (i.e., CFI, TLI, RMSEA, AIC, and BIC). Although there was substantial overlap between the two‐factor and the network model, this pattern suggests that latent dimensions and network representations capture partly overlapping aspects of the observed associations among EMA mood items. In particular, the comparatively favorable fit indices of the network model support the usefulness of network approaches as a complementary framework for describing conditional associations among momentary affective states, rather than as definitive evidence for model superiority or within‐person dynamic processes.

### 4.2 Methodological Implications

The present findings have several methodological implications for the psychometric evaluation of EMA‐based affect measures. First, the ambiguity observed in factorial solutions highlights the limitations of relying exclusively on latent variable models when assessing momentary affective states, which are inherently dynamic, context‐dependent, and interrelated. Traditional indices such as Cronbach's alpha and factor loadings may therefore provide only a partial picture of measurement quality in intensive longitudinal designs. In contrast, network approaches allow for the description of direct conditional associations between affective states and provide additional information about the relative connectedness and centrality of individual items within the observed affect network. Second, the generally modest convergence with retrospective questionnaires underscores that EMA measures should not be evaluated primarily in terms of redundancy with trait‐based instruments, but rather in terms of their ability to capture within‐person variability and short‐term affective processes. Together, these considerations suggest that future EMA research may benefit from combining factorial and network‐based approaches to obtain a more comprehensive understanding of scale structure, item functioning, and the observed organization of momentary psychological experiences.

### 4.3 Limitations and Strengths

Several limitations of this study should be acknowledged. First, the multilevel nature of EMA data posed important methodological challenges. Our analyses primarily described the pooled (marginal) structure of the EMA items across all assessments and therefore did not disentangle within‐person covariation (momentary change processes) from between‐person differences (stable individual tendencies). Furthermore, repeated observation from the same participants could appear in both EFA and CFA subsets, although originating from different assessment time points. This may reduce the statistical independence of the holdout sample and potentially inflate the apparent stability of the factor structure. As a result, the EFA/CFA split should be regarded as a structural consistency check rather than as a fully independent validation of the factor structure. Additionally, EMA data were analyzed across both study arms, such that group‐specific response patterns or intervention‐related reactivity may have influenced the results. Because assessments followed regular prompting schedules and allowed for self‐initiated entries, some degree of reactivity and non‐random sampling cannot be ruled out (Myin‐Germeys et al. 2018). Future work should apply multilevel psychometric approaches (e.g., multilevel factor models, within‐ and between‐person network models, and time‐series methods) to more directly capture within‐person temporal dynamics. Second, our sample was relatively homogeneous in terms of age, educational background, and cultural context, and consisted exclusively of young and stressed Swiss university students. Moreover, participants with self‐reported mental disorders were excluded, but these exclusions were not verified through clinical interviews. Together, these factors limit the generalizability of the findings, particularly to more diverse or clinical populations. In addition, the assessment period was relatively short (1 week), which may have constrained the range of affective variability and limited the observation of longer‐term mood dynamics. Third, the present study examined a single brief EMA mood item set within a specific study protocol. Although the brevity of the scale is a strength in terms of feasibility, the limited number of items restricts content coverage and may constrain the representation of more nuanced affective states. Furthermore, all data relied on self‐report, which may be subject to reporting biases. Lastly, although we examined convergent associations with established psychometric measures, the external validation is inherently limited by conceptual differences between self‐rating questionnaires and EMA‐based assessment of transient affective states. As such, weak or modest correlations should be interpreted with caution. Therefore, future studies should consider using validation measures specifically designed to assess short‐term or momentary states, as well as predictive or criterion‐based outcomes, to further evaluate the validity and utility of brief EMA mood scales.

At the same time, several aspects strengthen this study. In particular, the EMA mood scale demonstrated good internal consistency, and its structure was examined comprehensively using both parametric exploratory factor analysis and non‐parametric network analysis (Christensen and Golino 2021). This combined approach allowed for a nuanced evaluation of the item set from complementary methodological perspectives. Given the nested and intensive longitudinal nature of EMA data, network analysis offers a particularly useful way to explore direct relationships between affective states that may not be adequately captured by traditional latent variable models. Together, these methodological choices strengthen the interpretability of the findings and underscore the value of integrating factorial and network approaches in the psychometric evaluation of EMA measures.

### 4.4 Implications for Digital Interventions and Future Directions

Our analyses revealed patterns of associations among affective states that suggest the present 11‐item EMA mood set may be capable of differentiating between multiple aspects of mood in a stress‐enriched non‐clinical student sample. This characteristic may be particularly relevant for Internet‐based and digital interventions, where repeated administration and lengthy self‐report questionnaires often lead to low user engagement and compromised data quality. Brief EMA assessments offer a promising alternative by providing ecologically valid, low‐burden measures that are easier to integrate into daily life. As such, EMA‐based mood assessments may enhance both ecological validity and sensitivity of outcome measurement in digital intervention contexts. Future studies should explore how brief EMA measures, such as the present mood item set, can be embedded in Internet‐based treatment contexts to improve monitoring, personalization, and prediction of therapeutic change. In addition, future research is needed to explore the performance at individual and collective level of brief EMA mood measures across longer time periods, diverse situational contexts, and clinical populations, building on the present findings and extending their generalizability.

## 5 Conclusion

The present study suggests that a brief 11‐item smartphone‐based EMA mood scale may capture coherent and meaningful patterns of momentary affect in everyday life. By accounting for differences between self‐remembering and self‐experiencing (Conner and Barrett 2012; Zajchowski et al. 2017), this brief EMA‐based mood measure may be particularly suitable for assessing momentary affective states with minimal retrospective bias. The item set showed good internal consistency and a reasonably interpretable structure, with convergent associations to established measures largely in line with conceptual expectations, supporting its potential utility for assessing transient mood states rather than trait‐like psychopathology. Across factorial and network analyses, affective experiences appeared to be organized along two broad dimensions of positive and negative affect, with possible differentiation within the negative domain into anxious/distressed and depressive factors. Importantly, network models showed comparatively favorable fit indices relative to the tested factorial models and offered a parsimonious representation of the observed associations among EMA items, highlighting the interconnected structure of the observed associations among EMA items. Together, these findings support the potential relevance of network approaches for EMA research and suggest that brief EMA mood measures could prove useful for monitoring affective processes in future clinical studies and digital intervention contexts.

## Author Contributions

**Steffi Weidt**: writing – review and editing. **Erich Seifritz**: writing – review and editing, resources. **Judith Rohde**: conceptualization, methodology, investigation, writing – review and editing, writing – original draft, project administration. **Marta A. Marciniak**: conceptualization, writing – review and editing. **Stephanie Homan**: writing – review and editing, writing – original draft, visualization. **Stephan T. Egger**: conceptualization, methodology, formal analysis, investigation, writing – original draft, writing – review and editing, supervision, visualization.

## Funding

The authors have nothing to report.

## Ethics Statement

Ethical approval was obtained from the Faculty of Arts and Social Sciences Ethics Committee of the University of Zurich (No. 20.4.24). The authors assert that all procedures contributing to this work comply with the ethical standards of the relevant national and institutional committees on human experimentation and with the Helsinki Declaration, as revised in 2013.

## Patient Consent Statement

All participants provided electronic informed consent.

## Conflicts of Interest

The authors declare that they have no known competing financial interests or personal relationships that could have appeared to influence the work reported in this paper.

## Clinical Trial Registration

The trial was retrospectively registered on ClinicalTrials.gov (NCT05617248).

## Supporting information




**Supplementary Material**: brb371598‐sup‐0001‐SuppMat.docx

## Data Availability

The data that support the findings of this study are available from the corresponding author upon reasonable request.
