# Supplementary material for: Psychometric Evaluation of Ecological Momentary Assessment Items for Mood in a Non‐Clinical Sample
Source: Brain Behav. 2026 Jul 14;16(7):e71598. doi: 10.1002/brb3.71598 (PMC13366398; doi:10.1002/brb3.71598)
Supplement: Supplementary file 1 — Supplementary Material: brb371598‐sup‐0001‐SuppMat.docx [file BRB3-16-e71598-s001.docx]

Supplementary Material for «Psychometric evaluation of ecological momentary assessment items for mood in a non-clinical sample»

This file includes:

- Supplementary Figures 1-3
- Supplementary Tables S1-S2

# Figures

| **Supplementary Figure 1** |
| --- |
| *Proportion of Completed Items*  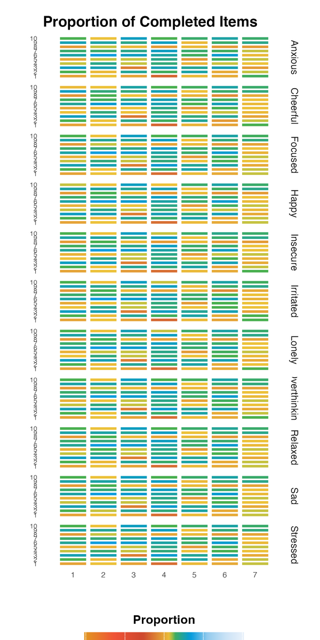 |
| *Notes*. Proportion of completed item responses across EMA assessment periods for each item. The figure shows the percentage of completed responses for 11 affect-related items over time. Higher values indicate a higher proportion of completed item responses. Differences across items illustrate variation in completion patterns across the assessment period. |

| **Supplementary Figure 2** |
| --- |
| *EMA Mood Variability Over Time*   |
|  |
| *Notes.* Ecological momentary assessment (EMA) mood ratings over time. Thin lines represent individual participant trajectories, while thick lines indicate the group mean. Positive affect items (cheerful, happy, relaxed, focused) and negative affect items (overthinking, irritated, anxious, insecure, lonely, sad, stressed) are displayed across days to illustrate within-person variability and temporal patterns. Overall, this item set shows substantial variability both between and within participants, reflecting dynamic fluctuations in mood over time. |

| **Supplementary Figure 3** |
| --- |
| *Centrality Indices of the EMA*  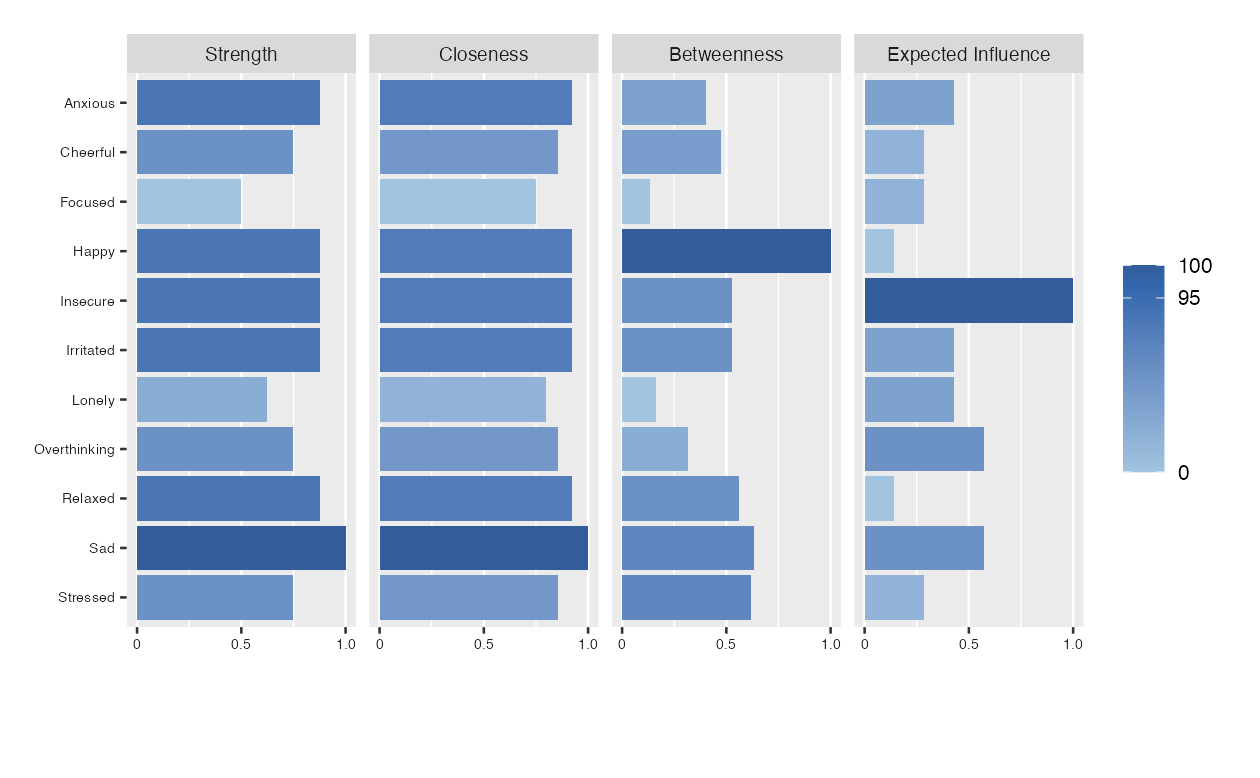 |
|  |
| *Notes.*Centrality measures for each node in the ecological momentary assessment (EMA) affect network. Strength indicates the sum of absolute edge weights connected to a node. Closeness reflects how close a node is to all others in the network. Betweenness represents the number of shortest paths passing through a node. Expected influence summarizes the signed sum of edge weights connected to a node and reflects the overall pattern of positive and negative associations with other nodes. Nodes are labelled according to affective items. Key patterns include high strength and closeness for “sad”, high betweenness for “happy”, and high expected influence for “insecure”. |

# Tables

**Supplementary Table S1**

*Concordance Correlation Coefficients and Bland–Altman Agreement Between Aggregated EMA Mood Scores and Baseline Questionnaires*

|  | Correlation Coefficient |  |  | Bland Altman |  |
| --- | --- | --- | --- | --- | --- |
|  | Value | Upper CI | Lower CI | Observations in Upper Limit | Observations in Lower Limit |
| BDI | 0.06 | 0.25 | -0.15 | 1 (1.1%) | 1 (1.1%) |
| STAI trait | 0.14 | 0.33 | 0.06 | 0 (0.0%) | 0 (0.0%) |
| STAI state | 0.11 | 0.30 | -0.09 | 0 (0.0%) | 0 (0.0%) |
| PANAS positive | 0.15 | 0.33 | 0.07 | 0 (0.0%) | 0 (0.0%) |
| PANAS negative | 0.09 | 0.28 | -0.11 | 1 (1.1%) | 0 (0.0%) |
| GSE | 0.05 | 0.23 | -0.16 | 1 (1.1%) | 0 (0.0%) |
| PSS | 0.15 | 0.34 | -0.06 | 1 (1.1%) | 0 (0.0%) |

**Supplementary Table S2**

*Fit Indices of the Factorial and Network Models of the EMA*

| Fit Index | χ² (df), p | CFI | TLI | AIC | BIC | RMSEA |
| --- | --- | --- | --- | --- | --- | --- |
| Cut-Off Criteria | - | > 0.95 | > 0.95 | lowest | lowest | < 0.06 |
| One Factor | *4734 (df = 44); P* < .001 | 0.82 | 0.77 | 172’788.48 | 172’930.29 | 0.15 (0.14-0.16) |
| Two Factor | *1989 (df = 43); P* < .001 | 0.93 | 0.90 | 170’045.45 | 170’193.71 | 0.09 (0.08-0.10) |
| Three Factor | *1222 (df = 41); P* < .001 | 0.95 | 0.94 | 169’282.32 | 169’443.47 | 0.08 (0.07-0.09) |
| Network Model | *57 (df = 39); P* = .03 | 0.99 | 0.99 | 168’114.35 | 168’417.32 | 0.01 (0.00-0.02) |

*Notes.* CFI: Comparative Fit Index; TLI: Tucker-Lewis Index; AIC: Akaike Information Criterion; BIC Bayesian Information Criterion; RMSEA: Root Mean Square Error of Approximation.
